# Validation of the Turkish version of the Chronic Stress Scale: assessing social role-related stressors and their impact on psychopathology

**DOI:** 10.3389/fpsyg.2024.1479845

**Published:** 2024-12-11

**Authors:** Hale Yapici Eser, Defne Ertuna, Merve Yalcinay-Inan, Imren Kurt Sabitay, Muhammed Balli, Can Misel Kilciksiz, Mehmet Utku Kucuker, Ozge Kilic, A. Cenk Ercan, Oya Guclu, Ömer Aydemir

**Affiliations:** ^1^Koç University School of Medicine, Istanbul, Türkiye; ^2^Koç University Graduate School of Health Sciences, Istanbul, Türkiye; ^3^Koç University Hospital, Istanbul, Türkiye; ^4^Department of Psychiatry, Başakşehir Çam Sakura City Hospital, Istanbul, Türkiye; ^5^Grossman School of Medicine, New York University, New York, NY, United States; ^6^Department of Psychiatry, Bezmialem Vakıf University, Istanbul, Türkiye; ^7^Department of Psychiatry, Manisa Celal Bayar University, Manisa, Türkiye

**Keywords:** stress, social role, anxiety, depression, Türkiye

## Abstract

**Introduction:**

Chronic social-role-related stress plays a crucial role in the development and progression of mental and medical disorders, making it an important factor to consider. This study aimed to translate and validate The Chronic Stress Scale (CSS) adapted by Turner for a Turkish population and explore its role in depression, anxiety, and perceived stress.

**Methods:**

A total of 524 participants (mean age 31.59 years, 68% women) were recruited from Koç University and Basaksehir Cam Sakura City Hospitals, including 260 from the general population and 264 with depressive or anxiety disorders. The 51-item CSS was translated into Turkish and validated through reliability and validity analyses, including Cronbach’s alpha, exploratory factor analysis, and correlations with the Beck Depression Inventory (BDI), Beck Anxiety Inventory (BAI), and Perceived Stress Scale (PSS-14).

**Results:**

The Turkish CSS showed good internal consistency (Cronbach’s alpha = 0.90) and identified 13 dimensions of chronic stress (partner, children, work, loneliness, finances, workload, debt, relationship inoccupancy, family health, residence, family, ex-partner, and others). Dimensions were named based on the content of the items included. Significant correlations were found between CSS and BDI (*r* = 0.611, *p* < 0.001), BAI (*r* = 0.558, *p* < 0.001), and PSS-14 (*r* = 0.222, *p* < 0.001). Discriminant validity revealed significant score differences between clinical and general populations.

**Conclusion:**

The Turkish CSS is a reliable and valid tool for assessing chronic social role-related stressors, supporting its use for both research and practice.

## 1 Introduction

Stress is a factor that affects individuals from the prenatal period through old age. Any physical, psychological, or environmental change that requires adaptation can serve as a stressor, with different types of stressors becoming more prevalent at various stages of life (Albayrak et al., 2024). Exposure to stress plays a significant role in the development, onset, and progression of mental and medical disorders. Stressors may be transient, recurrent, or persistent, the latter being referred to as chronic stress. Chronic stress is associated with an increased prevalence of depression (Richter-Levin and Xu, 2018; Cathomas et al., 2019), anxiety (Daviu et al., 2019), sleep disorders such as chronic insomnia (Kalmbach et al., 2018), and substance use disorders (Torres-Berrío et al., 2018). Furthermore, chronic stress can contribute to the onset of Alzheimer’s disease (Justice, 2018), trigger migraine headaches (Maleki et al., 2012; Yapıcı-Eser et al., 2018), and has been linked to some cancers, such as breast cancer (Chiriac et al., 2018; Yapıcı-Eser and Özyaprak, 2018) and cardiovascular diseases (Steptoe and Kivimäki, 2012).

Stressors such as job loss or divorce can significantly disrupt psychological functioning. However, even when an individual’s functionality remains unchanged, cognitive, emotional, and biological responses to stressors may lead to alterations in resilience capacity or adaptation (Albayrak et al., 2024; Crosswell and Lockwood, 2020), along with chemical and neurobiological alterations in the human body. Understanding the neurobiological mechanisms underlying stress resilience is crucial for elucidating individual differences in stress response, which can inform the development of new therapies for stress-related disorders such as major depressive disorder (MDD) and post-traumatic stress disorder (PTSD) (Cathomas et al., 2019).

For instance, firefighters who work in high-risk environments often exhibit lower levels of anxiety, depression, and stress compared to urban policemen and office employees, demonstrating higher emotional stability without differences in psychological wellbeing (Tommasi et al., 2021). These findings suggest that resilience acts as a protective factor, mitigating the adverse psychological effects of chronic stress.

Furthermore, systematic reviews indicate that stress exposure and resilience dynamically interact, exerting a significant influence on overall wellbeing (Li and Hasson, 2020).

Moreover, gender can significantly influence stress-coping mechanisms, empathy, biological stress responses, and resilience (Tommasi et al., 2023). Therefore, when conducting research on stress and resilience, it is essential to account for the role of sex and gender to ensure accurate and meaningful results. To study resilience and vulnerability, better documentation and investigation of the exposed stressors are necessary to understand biopsychosocial dynamics.

Among the various stressors, childhood life adversities are among the most extensively studied stressors, commonly assessed using self-report scales (Grummitt et al., 2021). However, it is also crucial to measure adult social role-related stressors, as they can significantly impact wellbeing and trigger psychiatric and other medical disorders, even in individuals with positive childhood experiences (McEwen, 2017). Measuring adult life stress is challenging due to variations in the type, duration, and timing of stressors and cultural differences in their perception. Life stressors are diverse, and not all events or triggers are distinctly separate. Some events can trigger a cascade of related events, while others may be so closely related that they appear redundant. Additionally, certain events or triggers may recur or persist over time. Therefore, stressors can be classified as acute, episodic, or chronic (Avison and Gotlib, 1994).

Moreover, adult life stressors may include a wide range of experiences, such as the death of a loved one, divorce, a demanding job, or caregiving for a family member with a disease such as Alzheimer’s. Social expectations may also induce different social role stressors. Stressors are not limited to past events but may extend over time through related experience and anticipation.

Chronic stress is characterized by actual or anticipated prolonged stressors that last for a significant period of time and require time to resolve and cope (Albayrak et al., 2024; Ross et al., 2017). The time for resolution of chronic stressors may be unpredictable, and sustained threat anticipation and coping activity may be provoked.

Chronic stressors may also include threats, complexity, uncertainty, lack of rewards, unmet expectations, demands, and structural constraints, such as limited access to opportunities (Wheaton et al., 2013, p. 304). Improved measurement of chronic adult life stressors is needed to assess their effects on wellbeing and resilience development. The Turner Chronic Stress Scale (CSS) is designed to assess long-term, persistent stressors related to social roles in adults. Unlike event-based stress measures, the CSS focuses on ongoing life circumstances, such as the experience of living as a divorced individual, rather than the divorce itself. It evaluates stress related to social role occupancy and inoccupancy, role-defining strains, as well as ambient, more diffuse, and general strains.

Additionally, the CSS captures non-events, like the absence of expected social role changes (e.g., not getting married or promoted). While the scale includes subjective reports, it aligns with individual perceptions and the significance of social roles in their lives. For instance, the psychological impact of not affording a desired home can vary greatly depending on personal expectations, yet the stress experienced may be similar. The 51-item CSS allows for both a cumulative stress score and a separate evaluation of stress dimensions related to different social roles. Its comprehensive assessment across multiple life domains—work, health, and relationships—makes it unique in measuring chronic social role-related stress in adults.

Given its potential to explore associations between social roles and psychiatric outcomes, validating and using the CSS in different languages is crucial. Despite the availability of some validated Turkish scales for evaluating some dimensions of social roles, such as occupational stress, there is currently no validated Turkish scale comparable to the CSS, which evaluates many dimensions of social stress. Having a measure for social role-related stressors can help researchers working on stress and resilience and clinicians who want to evaluate the dynamic changes of stressors in their patients’ lives. Assessing the biopsychosocial formulation is key in evaluating patients, and social role stressors are important factors to follow and document (Gilbert, 2015). A scale that evaluates multiple dimensions of social roles can also provide valuable insights for therapeutic interventions and clinical assessments.

This study aimed to translate the 51-item Chronic Stress Scale (CSS) into Turkish and evaluate its psychometric properties—including reliability, factorial structure, and convergent, divergent, and discriminant validity—to develop a culturally appropriate tool for assessing social role-related stressors in Turkish-speaking populations. We hypothesized that the Turkish version of the CSS would demonstrate strong internal consistency and exhibit significant positive correlations with depression and anxiety measures, supporting its convergent validity. Additionally, we anticipated that cultural differences might result in a factorial structure that diverges from the original scale, reflecting unique stress patterns within the Turkish context.

## 2 Methods

In this study, first permission was obtained from Turner J. to develop the Turkish version of the CSS. The translation was conducted using back-translation and consensus development. Participants from psychiatry outpatient units and the general population were recruited from two hospitals. The reliability, factorial structure, and convergent, divergent, and discriminant validity of the scale were analyzed.

### 2.1 Procedures for the Turkish version of the Chronic Stress Scale

Permission was obtained from J.R. Turner to validate the Turkish version of the Chronic Stress Scale (CSS). The translation process involved two stages: initial translation by a psychiatrist and psychologist, comparison and consensus.

The initial translation stage of the CSS was completed by a doctorate-level psychiatrist and a psychologist. The second stage consisted of a comparison of the translations between the psychiatrist and psychologist until a consensus was reached between them. Later, a native English speaker back-translated the Turkish version, ensuring consistency with the original. Finally, a second researcher then back-translated it again for further validation. The final translated version of the CSS was given to eight medical students, and this helped refine the final Turkish version, as provided in Appendix 1.

### 2.2 Participants

This study included 524 participants from clinical and general population groups who were recruited from two hospitals. The first dataset was drawn from a larger study at Koç University Hospital, targeting the validation of multiple scales (Eser et al., 2020a; Eser et al., 2020b). For this study, only patients with fully valid data for CSS and other scales mentioned in the methods section were included from the larger dataset recruited (*n* = 296). Patients with a diagnosis of a depressive disorder or anxiety disorder formed the clinical sample. Participants were recruited via advertisements and hospital waiting lounges, with inclusion criteria of age 18–65, literacy, and sufficient educational capacity. The exclusion criteria included schizophrenia spectrum disorders, bipolar I, dementia, history of head trauma, substance intoxication, or medical conditions affecting cognition.

Schizophrenia and bipolar I disorder were excluded, as the study focused on internalizing disorders such as depression and anxiety disorders, which are the main disorders associated with chronic stress (Tafet and Bernardini, 2003; McEwen, 2017). A second dataset (*n* = 228) was collected from Başakşehir Çam Sakura City Hospital (*n* = 180) and Koç University Hospital (*n* = 48) between December 2022 and November 2023, ensuring an adequate sample size for the CSS, required to have at least 10 participants for each item of the CSS. The same inclusion and exclusion criteria were applied.

The data collection process was conducted by trained research assistants, with participants completing an online survey via Qualtrics. Informed consent was obtained, and participants did not receive compensation, though feedback on scale scores was provided. Those with elevated scores in the general population group received psychoeducation and were advised to seek psychiatric consultation if necessary. All procedures were approved by the Koç University local ethics committee and adhered to the Declaration of Helsinki.

### 2.3 Instruments

A sociodemographic data form generated by the authors was used to collect information about participants’ age, sex, educational status, income, marital status, lifetime and current psychiatric and physical diagnoses, and ongoing treatments.

**Chronic Stress Scale (CSS)**: CSS was adapted by Turner R. J. from the scale developed by Wheaton (1991, 1997) to evaluate the chronic social role-related stressors in people’s lives (Turner et al., 1995). The chronic stress scale includes a list of 51 items about common life conditions and situations (e.g., financial issues, work, marriage and relationship, parental, family, and social life). Here, the participant was instructed to respond to each item using a three-point Likert scale: “not true” (0), “somewhat true” (1), or “very true” (2). If a particular item references a social role that the participant does not currently hold (e.g., the participant is unemployed, and the item pertains to occupation), they were directed to select “not true. The total score was calculated by adding up all item scores.

**Beck Depression Inventory (BDI)**: To assess the depressive symptoms of participants, BDI, developed by Beck and Steer (1984), was employed. The inventory consists of 21 items designed to measure cognitive, affective, and vegetative symptoms of depression, with responses recorded on a 4-point Likert scale. Psychometric analyses of the Turkish form of BDI were performed by Hisli (1989).

**Beck Anxiety Inventory (BAI)**: It consists of 21 items designed to assess anxious symptoms, with responses provided on a 4-point Likert scale. Developed by Beck et al. (1988), the inventory has been validated in Turkish by Ulusoy (1993). Higher scores indicate higher anxiety symptoms, and scores range from 0 to 63.

**Perceived Stress Scale-14 (PSS-14)**: PSS-14 was developed by Cohen et al. (1983). The objective is to assess the level of recent stress experienced by individuals and their coping abilities. Eskin et al. (2013) developed the Turkish adaptation of this scale.

### 2.4 Statistical analysis

Descriptive statistics were calculated for demographic variables, including age, sex, education, income, and occupational status, as well as mean scores of self-reported psychometric scales. Age groups were defined based on Gao et al. (2022): young adults (18–35 years) transitioning from education to work and middle-aged to older adults (36–65 years). Quality control checks were performed on clinical measures. Chi-square tests identified demographic differences between patient and healthy population groups. Reliability analysis included Cronbach’s alpha, item-total score correlation, and Cronbach’s alpha coefficient if an item was deleted. Item-total correlation was expected to be between 0.20 and 0.80. Confirmatory factor analysis (CFA) with the previously suggested dimensions of the scale (PhenX toolkit, 2024) was conducted using Jamovi (version 2.4) to evaluate the fit of the proposed model, with fit indices including χ^2^, RMSEA, CFI, and TLI used to assess model adequacy. Afterward, the Kaiser-Meier-Olkin and Bartlett’s tests were used to measure sampling adequacy for factor analysis. Exploratory factor analysis was performed as principal component analysis with varimax rotation with 25 iterations, and factors with an eigenvalue greater than 1 were included in the analysis. Another CFA analysis was conducted also with the resulting factors. Finally, Akaike Information Criterion (AIC) analyses using Jamovi were conducted to compare the fit of three models: the unidimensional model for CSS, the original dimensional model, and the Turkish dimensional model based on the factor analysis. AIC is a widely used metric for model comparison, with lower AIC values indicating better model fit while accounting for model complexity (Akaike, 1987).

Convergent and divergent validity of CSS were assessed using Pearson correlation between CSS and other clinical measures (BDI, BAI, PSS-14). The data was checked for normality analysis with the Shapiro-Wilks test. Discriminant validity was evaluated with the Mann–Whitney U-test, comparing healthy and clinical groups. A *p*-value of <0.005 is considered significant due to multiple comparisons. All analyses were conducted for both the total CSS score and its dimensions.

## 3 Results

### 3.1 Study sample

The mean age of the participants was 31.59 ±12.1 years (min = 18; max = 65). Of the 524 participants, 356 were women (67.9%), and the remaining 168 were men (32.1%). The participants’ sociodemographic characteristics are given in Supplementary Table 1 and the sociodemographic and health-related variables of the clinical and general population groups are given in Supplementary Table 2. The BAI scale’s mean was 17.49 ± 13.81 (min = 0, max = 63). For the BDI scale, the mean was 16.42 ± 11.81 (min = 0, max = 52), and for the PSS-14 scale, the mean was 31.42 ± 7.44 (min = 0, max = 54). For the control group, the skewness analysis conducted for BDI, BAI, PSS-1,4, and CSS total score yielded the following results: 1.50 (SE = 0.15), 1.52 (SE = 0.15), −0.47 (SE = 0.15), 0.99 (SE = 0.15) respectively. The kurtosis analysis conducted for BDI was 2.42 (SE = 0.30), for BAI 2.62 (SE = 0.30), for PSS-14 2.13 (SE = 0.30) and for CSS 1.11 (SE = 0.30). For the clinical group, the skewness analysis conducted for BDI, BAI, PSS-14 and CSS total score yielded the following results; 0.16 (SE = 0.15), 0.52 (SE = 0.15), −0.52 (SE = 0.15), 0.58 (SE = 0.15) respectively. The kurtosis analysis conducted for BDI was −0.52 (SE = 0.30), for BAI -0.55 (SE = 0.30), for PSS-14 1.47 (SE = 0.30) and for CSS 0.36 (SE = 0.30).

### 3.2 Reliability analysis and factor structure of the CSS

The Cronbach’s alpha coefficient was calculated to determine the internal consistency of the CSS and was found to be 0.90. The corrected item-total correlation coefficients ranged between 0.04 and 0.58.

The Cronbach’s alpha ranged between 0.895 and 0.90. Scale means if an item is deleted varied between 26.43 and 27.88. The corrected item-total correlation and Cronbach’s alpha if an item is deleted indicate good reliability for the CSS (Table 1).

**Table 1:** Internal reliability measures of the Turkish version of CSS.

	Scale mean if item deleted	Corrected item-total correlation	Cronbach’s Alpha if item deleted
CSS1	26.46	0.318	0.898
CSS2	27.10	0.444	0.897
CSS3	26.65	0.429	0.897
CSS4	27.40	0.459	0.897
CSS5	27.42	0.293	0.899
CSS6	27.53	0.333	0.898
CSS7	27.04	0.359	0.898
CSS8	26.56	0.274	0.899
CSS9	26.74	0.375	0.898
CSS10	27.57	0.369	0.898
CSS11	27.23	0.510	0.896
CSS12	26.95	0.442	0.897
CSS13	27.15	0.513	0.896
CSS14	27.27	0.397	0.898
CSS15	27.26	0.424	0.897
CSS16	27.36	0.418	0.897
CSS17	27.25	0.456	0.897
CSS18	27.51	0.429	0.897
CSS19	27.34	0.576	0.895
CSS20	27.36	0.494	0.896
CSS21	27.38	0.564	0.895
CSS22	27.45	0.538	0.896
CSS23	27.60	0.524	0.896
CSS24	27.63	0.430	0.897
CSS25	27.75	0.388	0.898
CSS26	27.36	0.195	0.900
CSS27	27.13	0.342	0.898
CSS28	27.74	0.249	0.899
CSS29	27.91	0.037	0.900
CSS30	27.20	0.555	0.895
CSS31	27.59	0.153	0.900
CSS32	27.77	0.307	0.899
CSS33	27.69	0.317	0.898
CSS34	27.67	0.304	0.899
CSS35	27.79	0.290	0.899
CSS36	27.63	0.152	0.900
CSS37	27.75	0.236	0.899
CSS38	27.52	0.374	0.898
CSS39	27.33	0.449	0.897
CSS40	27.61	0.367	0.898
CSS41	27.38	0.412	0.897
CSS42	27.21	0.352	0.898
CSS43	27.31	0.387	0.898
CSS44	27.12	0.509	0.896
CSS45	27.30	0.334	0.898
CSS46	27.53	0.156	0.900
CSS47	27.34	0.276	0.899
CSS48	27.75	0.291	0.899
CSS49	27.74	0.149	0.900
CSS50	27.47	0.318	0.899
CSS51	27.75	0.150	0.900

The confirmatory factor analysis indicated a significant chi-square test [χ^2^(1148) = 2,964, *p* < 0.001]. The RMSEA was 0.0549 (90% CI [0.0525, 0.0574]), suggesting a good model fit. However, the CFI (0.812) and TLI (0.791) were below the commonly accepted threshold of 0.90, indicating that while the model demonstrates an acceptable fit, there remains room for improvement.

Kaiser-Meyer-Olkin’s measure of sampling adequacy was 0.87, and Bartlett’s Test of Sphericity, a measure assessing whether the dataset is suitable for factor analysis, yielded a significant result (*p* < 0.001). Therefore, the results of the factor analysis for the 51 items of CSS were interpreted.

The factor analysis revealed 13 distinct factors named dimensions, explaining 61.55% of the variance (Table 2). When items loaded for each factor were investigated, names of each factor were defined as partner, children, work, loneliness, financial, workload, debt, relationship inoccupancy, family health, residence, family, ex-partner, and others. CSS items loaded for each factor/dimension are given in Supplementary Table 3. Item# 31 was not loaded under a factor significantly and had a low item-total item correlation in reliability analysis. The confirmatory factor analysis based on the dimension revealed a significant chi-square test [χ^2^(1097) = 2,544, *p* < 0.001]. The RMSEA was 0.0502 (90% CI [0.0476, 0.0527]), indicating a good fit. The CFI (0.849) and TLI (0.831), although again below the preferred threshold of 0.90, suggest that the model fit is approaching acceptability compared to the previous analysis. The Akaike Information Criterion (AIC) analysis revealed that the unidimensional model yielded an AIC value of 50,055, the original dimensional model produced an AIC of 45,039, and the Turkish dimensional model demonstrated the lowest AIC at 44,711.

**Table 2:** Factor loadings of the Turkish version of CSS.

CSS item number	CSS item	Factors												
		1	2	3	4	5	6	7	8	9	10	11	12	13
CSS19	Your partner does not understand you.	**0.849**	0.114	0.076	0.104	0.063	0.108	0.021	−0.075	0.062	0.051	−0.018	0.026	0.042
CSS21	You do not get what you deserve out of your relationship.	**0.811**	0.150	0.039	0.120	0.028	0.088	0.052	0.203	0.041	−0.012	−0.091	−0.153	0.113
CSS22	Your partner does not show enough affection.	**0.808**	0.156	0.023	0.066	0.025	0.052	0.087	0.221	0.030	−0.016	−0.023	−0.191	0.121
CSS20	Your partner expects too much of you.	**0.754**	0.097	0.089	0.124	0.007	0.109	0.076	−0.215	0.062	0.019	0.030	0.123	−0.045
CSS17	You have a lot of conflict with your partner.	**0.734**	0.110	0.021	0.023	0.110	0.088	−0.020	−0.161	0.123	0.009	0.075	0.070	0.001
CSS23	Your partner is not committed enough to your relationship.	**0.724**	0.162	0.075	0.090	0.035	−0.017	0.066	0.351	−0.019	−0.066	0.081	−0.160	0.119
CSS18	Your relationship restricts your freedom	**0.662**	−0.001	0.122	0.135	0.071	0.114	−0.020	−0.263	−0.030	0.096	0.039	0.236	−0.153
CSS24	Your sexual needs are not fulfilled by this relationship.	**0.616**	0.105	0.138	0.045	−0.014	0.000	0.051	0.063	0.041	−0.027	0.291	−0.018	−0.028
CSS25	Your partner is always threatening to leave or end the relationship.	**0.565**	0.042	0.115	−0.005	0.027	−0.024	0.001	0.199	−0.075	0.184	−0.026	0.110	0.109
CSS34	A child’s behavior is a source of serious concern to you.	0.106	**0.860**	0.028	0.016	−0.026	0.073	0.041	−0.019	0.079	0.083	0.021	0.024	−0.081
CSS33	You feel your children do not listen to you.	0.196	**0.808**	−0.004	0.031	−0.004	0.032	0.033	−0.048	0.032	0.056	0.015	0.055	0.011
CSS32	One of your children seems very unhappy.	0.107	**0.784**	0.037	0.002	0.062	0.079	−0.066	−0.006	0.061	0.043	0.089	0.064	−0.004
CSS35	One or more children do not do well enough at school or work.	0.071	**0.773**	0.092	0.063	−0.046	0.053	0.079	0.107	0.008	−0.024	−0.019	−0.003	−0.095
CSS36	Your children do not help around the house.	0.155	**0.661**	−0.066	−0.018	−0.043	−0.009	0.060	−0.286	0.013	−0.034	−0.030	0.036	0.176
CSS37	One of your children spends too much time away from the house.	0.100	**0.652**	0.048	0.080	−0.048	−0.073	0.021	−0.063	0.082	−0.109	−0.006	0.089	0.281
CSS12	Your job often leaves you feeling both mentally and physically tired.	0.082	−0.004	**0.744**	0.040	−0.039	0.288	0.046	0.037	0.043	−0.035	0.060	0.015	0.160
CSS15	Your work is boring and repetitive.	0.111	−0.027	**0.737**	0.135	0.118	0.021	−0.018	−0.013	0.036	−0.004	−0.012	0.080	0.048
CSS11	You want to change jobs or careers but do not feel you can.	0.154	0.143	**0.689**	0.267	0.124	0.019	−0.035	0.004	0.005	0.032	−0.005	−0.132	−0.023
CSS13	You want to achieve more at work, but things get in the way.	0.064	0.086	**0.644**	0.285	0.091	0.224	0.029	0.059	0.018	0.044	−0.039	−0.067	0.034
CSS14	You do not get paid enough for what you do.	0.076	0.037	**0.560**	−0.117	0.171	0.062	0.298	0.109	0.094	0.289	−0.082	0.058	−0.029
CSS10	Your supervisor is always monitoring what you do at work.	0.062	−0.024	**0.552**	−0.108	0.082	0.134	0.266	0.195	0.095	0.196	0.052	0.008	−0.071
CSS41	You do not have enough friends.	0.131	−0.054	0.187	**0.714**	0.046	−0.015	0.078	0.133	−0.033	0.038	0.012	0.039	−0.021
CSS30	You are alone too much.	0.242	0.071	0.100	**0.588**	0.127	0.185	−0.068	0.360	0.047	0.153	0.060	0.017	−0.023
CSS50	A long-term health problem prevents you from doing what you like.	0.059	0.204	0.036	**0.564**	0.035	0.024	0.120	−0.127	0.203	−0.024	−0.060	−0.077	−0.019
CSS39	You have to go to social events alone, and you do not want to.	0.186	0.141	0.133	**0.540**	0.070	−0.124	0.009	0.104	0.138	0.097	0.096	0.008	0.318
CSS40	Your friends are a bad influence.	0.024	0.005	0.194	**0.452**	−0.025	0.132	0.082	0.147	0.007	0.106	0.257	0.217	0.105
CSS43	You want to live farther away from your family.	0.080	−0.089	−0.007	**0.435**	0.302	0.346	−0.103	0.252	0.032	0.148	−0.036	−0.058	0.061
CSS2	There is too much pressure on you to be like other people.	0.120	−0.049	0.341	**0.379**	0.186	0.375	−0.131	0.156	−0.061	0.023	0.072	−0.147	−0.182
CSS7	You do not have enough money to take vacations.	0.034	−0.038	0.142	0.073	**0.825**	0.039	0.135	0.076	−0.057	0.014	0.065	0.034	0.050
CSS8	You do not have enough money to pay a home down.	−0.012	−0.091	0.089	0.000	**0.680**	0.138	0.152	0.106	−0.040	0.058	0.051	0.056	0.038
CSS4	You do not have enough money to buy the things you or your kids need.	0.173	0.078	0.145	0.108	**0.634**	0.045	0.301	0.063	0.071	0.039	−0.022	−0.046	0.038
CSS16	You are looking for a job and cannot find the one you want.	0.128	0.002	0.483	0.247	**0.495**	−0.084	−0.140	−0.006	−0.010	0.088	−0.120	0.063	−0.143
CSS1	You’re trying to take on too many things at once.	0.093	0.096	0.094	0.016	0.026	**0.718**	0.032	0.046	0.047	−0.022	0.005	−0.033	−0.024
CSS3	Too much is expected of you by others.	0.153	0.044	0.143	0.154	0.056	**0.676**	−0.010	0.022	0.134	0.094	0.021	0.070	−0.015
CSS9	You have more work to do than most people.	0.054	0.063	0.268	−0.088	0.129	**0.630**	0.191	−0.005	0.034	−0.002	0.105	−0.001	0.206
CSS6	Your rent or mortgage is too much.	0.088	0.094	0.105	0.042	0.288	0.047	**0.799**	−0.070	0.073	−0.027	0.044	−0.026	0.048
CSS5	You have a long-term debt or loan.	0.094	0.074	0.071	0.077	0.218	0.062	**0.798**	−0.138	0.077	−0.006	0.047	−0.046	−0.056
CSS31	You wish you could have children, but you cannot.	0.023	−0.191	0.064	0.260	−0.209	0.074	**0.**356	0.151	−0.046	0.336	−0.180	0.261	0.036
CSS26	You wonder whether you will ever get married.	0.041	−0.253	0.129	0.183	0.118	0.029	−0.089	**0.628**	−0.037	0.047	0.004	0.176	0.038
CSS27	You find it too difficult to find someone compatible with you.	0.057	−0.106	0.192	0.353	0.199	0.114	−0.174	**0.611**	−0.034	0.006	0.055	−0.018	−0.011
CSS48	You have a parent, a child, or a spouse or partner who is in very bad health and may die.	0.121	0.025	0.158	0.043	−0.037	0.049	0.089	0.038	**0.828**	0.010	0.027	0.066	−0.027
CSS47	Someone in your family or a close friend has a long-term illness or handicap.	−0.008	0.133	0.058	0.195	0.062	0.104	−0.020	−0.058	**0.675**	0.098	0.245	−0.026	−0.079
CSS51	You take care of an aging parent almost every day.	0.063	0.131	−0.061	−0.004	−0.059	0.089	0.095	−0.018	**0.640**	0.021	−0.245	−0.048	0.293
CSS45	The place you live is too noisy or too polluted.	0.058	0.067	0.202	0.143	0.088	0.006	−0.030	−0.003	0.064	**0.813**	0.108	−0.049	0.024
CSS44	You would like to move, but you cannot.	0.248	−0.010	0.055	0.293	0.363	0.214	0.029	0.046	0.171	**0.461**	−0.060	−0.150	0.063
CSS49	Someone in your family has an alcohol or drug problem.	0.129	0.041	0.010	0.053	0.026	0.010	−0.020	−0.006	0.096	−0.055	**0.683**	−0.009	−0.115
CSS46	Your family lives too far away.	0.034	0.008	−0.099	0.037	0.031	0.140	0.105	0.077	−0.104	0.192	**0.619**	−0.006	0.328
CSS29	You do not see your children from a former marriage as much as you would like.	−0.016	0.155	−0.002	−0.022	0.023	−0.005	−0.077	0.042	−0.016	−0.049	−0.028	**0.786**	0.069
CSS28	You have a lot of conflict with your ex-spouse.	0.140	0.277	−0.093	0.096	0.109	−0.005	0.130	0.394	0.101	−0.047	0.082	**0.461**	−0.147
CSS38	You feel like being a housewife is not appreciated.	0.298	0.319	0.021	0.044	0.216	0.045	−0.117	0.053	0.107	0.026	−0.041	0.019	**0.531**
CSS42	You do not have time for your favorite leisure time activities.	0.077	−0.102	0.277	0.249	−0.022	0.337	0.071	−0.102	0.030	0.046	0.166	0.077	**0.431**

Bold values indicate the factor item is loaded under.

### 3.3 Convergent and divergent validity of CSS: correlations of CSS scores with BDI, BAI and PSS-14

**Figure 1:**
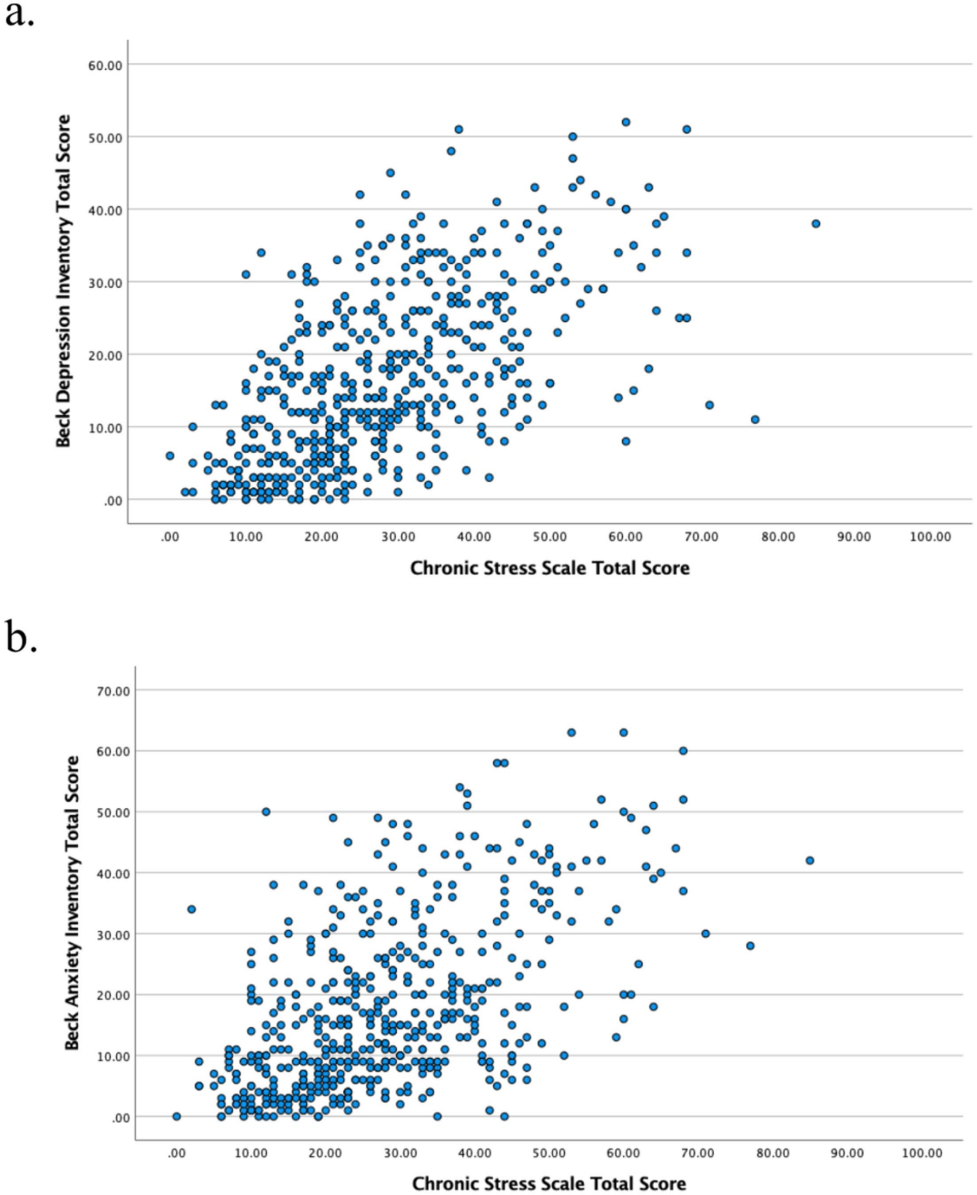
Correlation of CSS total score with Beck Depression Inventory (BDI) (a) and Beck Anxiety Inventory (BAI) (b) total scores. CSS total score showed a strong correlation with BDI (*r* = 0.61, *p* < 0.001) and a moderate correlation with BAI (*r* = 0.558, *p* < 0.001).

When CSS was examined for its correlation with BDI, BAI, and PSS-14 scales, the highest correlation was found between CSS and BDI (*r*: 0.61, *p*: <0.001) and BAI (*r*: 0.56, *p*: <0.001) (Table 3; Figure 1). CSS was also weakly but significantly correlated with PSS-14 (*r*: 0.22, *p*: <0.001). PSS-14 also showed a weak correlation with BDI (*r* = 0.24, *p* < 0.001) and BAI (*r* = 0.28, *p* < 0.001, Supplementary Figure 1).

**Table 3:** Convergent and Divergent Validity of the Turkish version of CSS and its factors with Beck Anxiety Inventory, Beck Depression Inventory, and Perceived Stress Scale-14.

		CSS	BAI	BDI	PSS-14
CSS Total Score	P.c.c.	1	0.558**	0.611**	0.222**
	p		<0.001	<0.001	<0.001
Factor 1 – Partner	P.c.c.	0.698**	0.322**	0.343**	0.085
	p	<0.001	<0.001	<0.001	0.051
Factor 2 – Children	P.c.c.	0.380**	0.159**	0.098*	0.004
	p	<0.001	<0.001	0.025	0.926
Factor 3 – Work	P.c.c.	0.681**	0.328**	0.423**	0.146**
	p	<0.001	<0.001	<0.001	<0.001
Factor 4 – Loneliness	P.c.c.	0.730**	0.563**	0.667**	0.231**
	p	<0.001	<0.001	<0.001	<0.001
Factor 5 – Financial	P.c.c.	0.562**	0.323**	0.378**	0.137**
	p	<0.001	<0.001	<0.001	0.002
Factor 6 – Workload	P.c.c.	0.549**	0.328**	0.342**	0.227**
	p	<0.001	<0.001	<0.001	<0.001
Factor 7 – Debt	P.c.c.	0.386**	0.192**	0.189**	−0.004
	p	<0.001	<0.001	<0.001	0.926
Factor 8 – Relationship Inoccupancy	P.c.c.	0.375**	0.263**	0.328**	0.212**
	p	<0.001	<0.001	<0.001	<0.001
Factor 9 – Family Health	P.c.c.	0.375**	0.173**	0.147**	0.069
	p	<0.001	<0.001	<0.001	0.112
Factor 10 – Residence	P.c.c.	0.570**	0.389**	0.427**	0.138**
	p	<0.001	<0.001	<0.001	0.002
Factor 11 – Family	P.c.c.	0.256**	0.127**	0.113**	0.041
	p	<0.001	0.004	0.010	0.349
Factor 12 – Ex-Partner	P.c.c.	0.268**	0.097*	0.076	0.090*
	p	<0.001	0.026	0.081	0.039
Factor 13-Other	P.c.c.	0.538**	0.328**	0.278**	0.202**
	p	<0.001	<0.001	<0.001	<0.001

P.c.c., Pearson Correlation Coefficient; p, *p*-value; CSS, Chronic Stress Scale; BAI, Beck Anxiety Inventory; BDI, Beck Depression Inventory; PSS-14, Perceived Stress Scale-14. *p < 0.05, **p < 0.005.

For further analysis of each dimension, correlations between the total scores of each dimension (BDI, BAI, and PSS-14) were calculated. CSS dimension 4, which pertains to loneliness-related stressors, exhibited the strongest correlation with the BDI (*r*: 0.667, *p*: <0.001), while dimension 12, ex-partner-related stressors, showed the weakest correlation (*r*: 0.076, *p*: 0.081) with BDI. Similarly, CSS dimension 4, loneliness-related stressors, had the highest correlation with the BAI (*r*: 0.563, *p*: <0.001), while dimension 12, ex-partner-related stressors, again displayed the lowest correlation (*r*: 0.097, *p*: <0.026). Finally, in relation to the PSS-14, dimension 4, loneliness-related stressors, demonstrated the strongest correlation (*r*: 0.231, *p*: <0.001), whereas dimension 7, debt-related stressors, exhibited the weakest correlation (*r*: −0.004, *p* = 0.926, Table 3).

### 3.4 Discriminant validity of CSS

The Mann–Whitney U-test was used to assess differences in CSS, BDI, BAI, and PSS-14 scores between general and clinical population groups. Clinical groups reported higher levels of chronic stress, anxiety, and depression (Supplementary Table 4). Differences were significant in CSS dimensions 1 (partner), 3 (work), 4 (loneliness), 5 (financial), 6 (workload), 8 (relationship inoccupancy), and 10 (residence), but not in dimensions 2 (children), 7 (debt), 9 (family health), 11 (family), 12 (ex-partner), and 13 (other) (Supplementary Table 4). Age significantly influenced CSS dimensions, reflecting distinct social role stressors (Supplementary Table 5). Females reported higher stress related to children (factor 2) and factor 13, which includes being a housewife (Supplementary Table 6). Although overall CSS scores did not significantly differ by sex, females reported higher BDI, BAI, and PSS scores, independent of clinical group status (Supplementary Table 2). Those who reported a physical diagnosis in the family reported significantly higher family health factor scores (factor 9) compared to those who did not (1.29 ± 1.51 vs. 0.70 ± 1.18, respectively; <0.001), further validating the discriminative validity.

## 4 Discussion

In this study, which aimed to develop the Turkish version of the CSS, the scale demonstrated good internal consistency with a Cronbach’s alpha of 0.90. The corrected item-total correlation (CITC) coefficients ranged from 0.04 to 0.58. The lowest CITC was for item# 29, “You do not see your children from a former marriage as much as you would like,” assigned to dimension 12 in factor analysis, “Ex-partner.” This low correlation may be due to the small proportion of divorced individuals (17%) and the lack of data on the number of children, limiting analysis. The next lowest CITC was for item# 49, “Someone in your family has an alcohol or drug problem,” assigned to dimension 11 in factor analysis, “family,” likely reflecting the low number of participants with this issue. The other items with very weak CITC (26, 36, 46, and 51) were kept in the Turkish form of CSS and not removed from total score calculations or factorial analysis, as they still contribute to understanding stress dimensions significantly with well-explained factor loadings. Item# 31 both had a weak CITC and also low factor loading. Therefore, it has not been assigned to any factors/ dimensions, and we suggest that it can be used separately from the factors. Moreover, CSS can be used as a 50-item scale without involving item #31. This approach has not been preferred here as item# 31 can be a significant stressor for some populations.

The highest CITC of 0.576 was found for item 19, “Your partner does not understand you,” in dimension 1, “Partner-related stressors.” Satisfaction with a close relationship is a significant factor for resilience and mental and physical wellbeing (Milrad et al., 2019). Lack of understanding can lead to significant stress due to miscommunication and unresolved conflict. Recent research shows that poor communication and empathy deficits are strong predictors of relationship stress and dissatisfaction (Overall and McNulty, 2017). Moreover, a dyadic understanding of coping can reinforce resilience in couples (Aydogan et al., 2021). Therefore, the high CITC value for this item reflects its strong association with chronic stress.

Exploratory factor analysis of the Turkish version of CSS revealed 13 dimensions of chronic stress, consistent with the original scale (Supplementary Table 3). However, item categorization differed slightly, with some factors, such as partner, relationship inoccupancy, ex-partner, and children, remaining similar, while others, like work, workload, and loneliness, were restructured. For example, family-related health problems emerged as a distinct factor, and some items shifted categories (e.g., item 2 to loneliness). For the work category, the Turkish version has two subdimensions for work and workload. Isolation and social life factors of the original scale are one category of “loneliness.” In our study, the health category was broken into segments where family-related health problems were presented as one factor. A family living far away (item 46) is not loaded under residence but under family. Research done on caregivers of the elderly has revealed that women in Türkiye are usually responsible for caregiving to older adults, which has its own psychological and economic consequences (Terkoğlu and Memiş, 2022). It is suggested that the sociodemographic variables of our participant pool be considered while considering the factorial structure of the Turkish version of the CSS. The stability of the factorial structure in other Turkish samples may need to be assessed. On the other hand, the AIC results indicate that the Turkish dimensional model offers the best fit among the three, balancing model complexity and explanatory power more effectively than the original and unidimensional alternatives.

The CSS strongly correlated with the BDI and BAI measurements, whereas the correlation of PSS with the BDI and BAI was weak. In the study by Wheaton, chronic social role-related stressors showed a higher correlation with psychopathology compared to event stressors (time-limited events or situations), daily hassles, and childhood life adversities (Wheaton, 1994). This finding underscores the importance of chronic social role-related stressors’ effect on psychopathology and that not all role stressors may present as perceived stress by the individuals. It is important to document these variables as they may be associated with resilience factors (Dumont and Provost, 1999; Sippel et al., 2015).

Dimensions 1 (partner), 2 (children), and 11 (family-related stressors) showed no correlation with perceived stress and weak links with BDI and BAI. Strong family ties are linked to lower depression, anxiety, and loneliness (Chen and Harris, 2019), as social and emotional support from family helps individuals cope, reducing isolation and providing practical aid. A strong sense of purpose from family also buffers against stress, enhancing resilience and wellbeing and lowering risks of depression and anxiety (Nelson et al., 2012). Our findings in this study are consistent with the existing literature on the topic, reinforcing the conclusions drawn.

Loneliness (dimension 4) strongly correlated with the CSS total score, BDI, BAI, and PSS-14, highlighting the mental health impact of social isolation (McGonagle et al., 2024). Loneliness diminishes social support, leading to chronic stress accumulation and negative thought patterns that worsen depression and anxiety. It can also trigger physiological changes, like increased cortisol and inflammation, deepening stress. Loneliness-related stressors were more common in the clinical population, reflecting higher isolation, relationship loss, and lack of social support. Social withdrawal and avoidance, often driven by fear of judgment or low motivation, contribute to this stress (Lim et al., 2020). Depression and anxiety can impair social skills, increase loneliness, as well as reduce social engagement and physical health, further limiting social activity (Hawkley and Cacioppo, 2010; Loades et al., 2020). Our findings are in accordance with the literature discussed.

Financial stressors (dimension 5) significantly correlated with BDI, BAI, and PSS, while debt-related stressors (dimension 7) had weaker effects. Financial stressors impact perceived stress due to their influence on economic stability and daily life. Chronic worry about basic expenses increases anxiety and depression (Simonse et al., 2022; Guan et al., 2022). Financial stress, especially unpredictable and urgent, may lead to more intense chronic stress, while debt stress, though significant, is often more predictable. Access to a loan or mortgage may decrease the individuals’ financial stress and increase their quality of life. Debt delinquency and financial capability may be the mediators of the stress reports (Xiao and Kim, 2022).

The study revealed significant differences in scores for depression, anxiety, perceived stress, and chronic stress between clinical and general population groups. The clinical group’s stressors related to partner, work, loneliness, finances, workload, and residence were notably higher. This heightened stress could be linked to cognitive and emotional impairments associated with mental disorders, as well as maladaptive coping strategies like avoidance and withdrawal, which may exacerbate work-related stress (Bianchi et al., 2015). Partner- and residence-related stressors were also higher in the clinical population. Mental illness can strain romantic relationships, leading to increased conflict and stress. Individuals with depression and anxiety may exhibit insecure attachment styles, resulting in heightened dependency and relationship stress (Mikulincer and Shaver, 2012). Furthermore, clinical conditions can impair financial management, leading to housing-related stress. These factors likely explain the clinical group’s higher levels of relationship- and housing-related stress.

A limitation of this study is that the convergent and divergent validity of the CSS was only tested using BDI, BAI, and PSS. Other measures, like the Life Events and Difficulties Schedule (LEDS), assess chronic stressors through structured interviews and contextual assessments but require trained interviewers and blind raters (Brown, 2002; Harkness and Monroe, 2016). The Stress and Adversity Inventory (STRAIN) is a self-administered interview measuring lifetime stress, but it may require computer assistance (Slavich and Shields, 2018). The Hassles and Uplifts Scale by DeLongis et al. (1988) measures daily stress but is influenced by affective status and does not assess chronic stress (Furman et al., 2024). The Daily Stress Inventory (DSI) by Brantley et al. (1987) focuses on daily stressors, not chronic ones, and responses may be subjective. Finally, the Holmes and Rahe (1967) scale lists stressful life events but does not capture the same stress exposures as the CSS. Unlike these tools, the CSS uniquely evaluates unmet expectations and diffuse, non-event stressors in social domains like work, health, and relationships, providing a comprehensive assessment of chronic stress in adults. Therefore, they have not been used for convergent validity analysis. Here, the test–retest reliability of the scale has not been conducted in this study. A pilot study to explore potential challenges has not been explored, but it may have provided valuable insights and helped refine the methodology. The socio-demographic data for the current number of children or having a debt was not collected as a variable. Future studies may benefit from analyzing their correlations with the participant responses. The general population group was not evaluated with a face-to-face interview for a possible psychiatric diagnosis. The participants’ responses may still be under affective bias, and the chronic stress factors reported by the participants were not validated by another informant.

Furthermore, the decision to exclude patients with schizophrenia and bipolar disorder from the study, along with the absence of a geriatric sample, may hinder the generalizability of the results. Future research on the topic involving a diverse group of participants might enhance the generalizability of the results. On the other hand, this study has many strengths, such as a large sample size and the inclusion of both clinical and general population groups with variations in sociodemographic variables, which may reflect the general cultural context of people living in Türkiye. Conducting the study across two different centers at various time points further supports the generalizability of the findings. Additionally, the study assessed the association between internalizing disorders and stress, achieving a high correlation score that validates the measurement capacity of the scale. Finally, this is the first study to assess the validity of CSS in another language.

## 5 Conclusion

This study successfully developed and validated the Turkish version of the Turner Chronic Stress Scale (CSS), demonstrating its reliability and validity in assessing chronic social role-related stressors in the Turkish population. The CSS’s ability to measure both role occupancy and inoccupancy, along with its emphasis on long-term, diffuse stressors, offers a comprehensive tool for understanding the complex nature of chronic stress in adults.

The strong correlations between CSS scores and measures of depression and anxiety highlight its relevance in clinical settings. However, the study also highlighted certain limitations, such as the absence of sociodemographic data on participants’ children or debts and the need for further validation across different patient demographics. Despite these limitations, the Turkish CSS stands out as a valuable instrument for assessing chronic stress and its impact on mental health, with potential applications in both research and clinical practice.

## Data Availability

The raw data supporting the conclusions of this article will be made available by the authors, without undue reservation.
