# Supplementary material for: Validation of the Turkish version of the Chronic Stress Scale: assessing social role-related stressors and their impact on psychopathology
Source: Front Psychol. 2024 Dec 11;15:1479845. doi: 10.3389/fpsyg.2024.1479845 (PMC11670873; doi:10.3389/fpsyg.2024.1479845)
Supplement: Supplementary file 1 [file Table_1.docx]

Supplementary Table 1. Sociodemographic characteristics of the participant group (n=524)

| **Sociodemographic characteristics** | | |
| --- | --- | --- |
|  |  | **Mean ± S.D.** |
| **Age** | | 31.59 ± 12.17 |
|  |  | **n (%)** |
| **Sex** | |  |
|  | **Female** | 356 (67.9%) |
|  | **Male** | 168 (32.1%) |
| **Marital Status** | |  |
|  | **Married** | 185 (35.3%) |
|  | **In a Relationship** | 118 (22.5%) |
|  | **Single** | 132 (25.2%) |
|  | **Divorced-Widowed** | 89 (17.0%) |
| **Working Status** | |  |
|  | **Unoccupied** | 92 (17.6%) |
|  | **Retired** | 45 (8.6%) |
|  | **Student** | 170 (32.4%) |
|  | **Occupied** | 217 (41.4%) |
| **Education Level** | |  |
|  | **Primary School** | 17 (3.2%) |
|  | **Middle School** | 30 (5.7%) |
|  | **High School** | 179 (34.2%) |
|  | **2-Year University** | 60 (11.5%) |
|  | **University** | 191 (36.5%) |
|  | **Post Graduate** | 47 (9.0%) |
| **Having a Physical Disorder** | | 149 (28.4%) |
| **Having a Psychiatric Diagnosis** | | 203 (38.7%) |
| **Having a Family Psychiatric Diagnosis** | | 163 (31.1%) |
| **Using Alcohol** | | 230 (43.9%) |
| **Using Tobacco** | | 227 (43.3%) |
| **Using Psychoactive Substance** | | 27 (5.2%) |
| **Using Non-Psychiatric Medicine** | | 140 (26.7%) |

Supplementary Table 2. Comparison of sociodemographic and health related variables of the clinical and general population groups

|  |  | **General Population (n=260)** | **Clinical Population (n=264)** | X2 | P |
| --- | --- | --- | --- | --- | --- |
| **Sex** | |  |  |  |  |
|  | **Female** | 66.53% (173) | 69.31% (183) | 0.465 | 0.495 |
|  | **Male** | 33.46% (87) | 30.68% (81) |  |  |
| **Age** | |  |  |  |  |
|  | **18-35** | 63.85% (166) | 70.45% (186) | 2.594 | 0.107 |
|  | **36-65** | 36.15% (94) | 29.55% (78) |  |  |
| **Marital Status** | |  |  |  |  |
|  | **Married** | 35.38% (92) | 35.23% (93) | 18.734 | <.001 |
|  | **In Relationship** | 26.54% (69) | 18.56% (49) |  |  |
|  | **Single** | 17.69% (46) | 32.58% (86) |  |  |
|  | **Divorced** | 20.38% (53) | 13.64% (36) |  |  |
| **Working Status** | |  |  |  |  |
|  | **Unoccupied** | 6.54% (17) | 28.41% (75) | 44.051 | <.001 |
|  | **Retired** | 9.62% (25) | 7.58% (20) |  |  |
|  | **Student** | 38.45% (100) | 26.52% (70) |  |  |
|  | **Occupied** | 45.38% (118) | 37.50% (99) |  |  |
| **Education Level** | |  |  |  |  |
|  | **Primary School** | 2.30% (6) | 4.17% (11) | 8.077 | 0.152 |
|  | **Middle School** | 5.38% (14) | 3.05% (16) |  |  |
|  | **High School** | 37.30% (97) | 6.06% (82) |  |  |
|  | **2-Year University** | 8.84% (23) | 14.02% (37) |  |  |
|  | **University** | 35.38% (92) | 37.50% (99) |  |  |
|  | **Post Graduate** | 10.77% (28) | 7.20% (19) |  |  |
| **Having a Physical Disorder** | |  |  |  |  |
|  | **Yes** | 24.62% (64) | 32.20% (85) | 3.700 | 0.054 |
|  | **No** | 75.38% (196) | 67.80% (179) |  |  |
| **Having a Psychiatric Diagnosis** | |  |  |  |  |
|  | **Yes** | 6.92% (18) | 70.08% (185) | 220.136 | <.001 |
|  | **No** | 93.08 (242) | 29.92% (79) |  |  |
| **Having Psychiatric Disorder in the Family** | |  |  |  |  |
|  | **Yes** | 22.69% (59) | 39.39% (104) | 17.050 | <.001 |
|  | **No** | 77.31% (201) | 60.61% (160) |  |  |
| **Using Alcohol** | |  |  |  |  |
|  | **Yes** | 43.08% (112) | 41.67% (110) | 1.071 | .301 |
|  | **No** | 53.85% (140) | 58.33% (154) |  |  |
| **Using Tobacco** | |  |  |  |  |
|  | **Yes** | 39.62% (103) | 46.97% (124) | 2.885 | .089 |
|  | **No** | 60.38 % (157) | 53.03% (140) |  |  |
| **Using Psychoactive Substance** | |  |  |  |  |
|  | **Yes** | 6.54% (17) | 3.79% (10) | 2.028 | .154 |
|  | **No** | 93.46% (243) | 96.21% (254) |  |  |
| **Using Non-Psychiatric Medicine** | |  |  |  |  |
|  | **Yes** | 24.62% (64) | 28.79% (76) | 1.165 | 0.280 |
|  | **No** | 75.38% (196) | 71.21% (188) |  |  |

|  |  |  |  |  |  |
| --- | --- | --- | --- | --- | --- |
|  | |  |  |  |  |

Supplementary Table 3. CSS factors (dimensions) and items for each factor

| **Factor/ Dimension** | **CSS Items** |
| --- | --- |
| 1 - Partner | 17, 18, 19, 20, 21, 22, 23, 24, 25 |
| 2 - Children | 32, 33, 34, 35, 36, 37 |
| 3 - Work | 10, 11, 12, 13, 14, 15 |
| 4 - Loneliness | 2, 30, 39, 40, 41, 43, 50 |
| 5 - Financial | 4, 7, 8, 16 |
| 6 - Workload | 1, 3, 9 |
| 7 - Debt | 5, 6 |
| 8 - Relationship Inoccupancy | 26, 27 |
| 9 - Family Health | 47, 48, 51 |
| 10 - Residence | 44, 45 |
| 11 - Family | 46, 49 |
| 12 - Ex Partner | 28, 29 |
| 13-Others | 38, 42 |

Note: Item 31 does not have a significant factor load under any dimension.

Supplementary Table 4. Comparison of the general population and clinical population participants for the CSS factors, CSS total score, BDI, BAI and PSS-14

| **Scale (Min-Max scores)** | **General Population (Mean ± S.D.)** | **Clinical Population (Mean ± S.D.)** | **p** |
| --- | --- | --- | --- |
| CSS Factor 1- Partner (0-18) | 3.26 ± 4.25 | 4.95 ± 4.97 | <0.001 |
| CSS Factor 2 - Children (0-12) | 1.08 ± 2.14 | 1.50 ± 2.77 | 0.433 |
| CSS Factor 3-Work (0-12) | 3.33 ± 2.89 | 4.94 ± 3.45 | <0.001 |
| CSS Factor 4-Loneliness (0-14) | 2.78 ± 2.58 | 5.44 ± 3.20 | <0.0001 |
| CSS Factor 5-Financial (0-8) | 2.94 ± 2.16 | 3.76 ± 2.32 | <0.001 |
| CSS Factor 6-Workload (0-6) | 3.65 ± 1.54 | 4.23 ± 1.59 | <0.001 |
| CSS Factor 7-Debt (0-4) | 0.83 ± 1.23 | 1.00 ± 1.32 | 0.069 |
| CSS Factor 8-Relationship Inoccupancy (0-4) | 1.22± 1.37 | 1.53 ± 1.36 | 0.004 |
| CSS Factor 9-Family Health (0-6) | 0.83 ± 1.23 | 1.08 ± 1.47 | 0.077 |
| CSS Factor 10-Residence (0-4) | 1.15 ± 1.25 | 1.75 ± 1.33 | <0.001 |
| CSS Factor 11-Family (0-4) | 0.67 ± 0.90 | 0.52 ± 0.93 | 0.007 |
| CSS Factor 12-Ex-Partner (0-4) | 0.17 ± 0.53 | 0.26 ± 0.68 | 0.092 |
| CSS Factor 13- Other (0-4) | 1.01 ± 0.99 | 1.25 ± 1.15 | 0.020 |
| CSS Total Score (0-102) | 23.23 ± 12.54 | 32.56 ± 14.49 | <0.001 |
| BDI (0-63) | 10.79 ± 9.84 | 21.96 ± 10.95 | <0.0001 |
| BAI (0-63) | 11.88 ± 10.63 | 23.02 ± 14.36 | <0.0001 |
| PSS-14 (0-56) | 30.23 ± 7.38 | 32.59 ± 7.33 | <0.001 |
| Note: CSS: Chronic Stress Scale, BDI: Beck Depression Inventory, BAI: Beck Anxiety Inventory, PSS-14: Perceived Stress Scale-14, Mann-Whitney U test | | | |

Supplementary Table 5. Comparison of the age groups of participants for the CSS factors, CSS total score, BDI, BAI and PSS-14

| **Scale (Min-Max scores)** | **Age 18-35 (Mean ± S.D.)** | **Age 36-65 (Mean ± S.D.)** | **p** |
| --- | --- | --- | --- |
| CSS Factor 1- Partner (0-18) | 3.66 ± 4.58 | 5.03 ± 4.81 | <0.001 |
| CSS Factor 2 - Children (0-12) | 0.47 ± 1.59 | 2.97 ± 3.08 | <0.0001 |
| CSS Factor 3-Work (0-12) | 4.38 ± 3.33 | 3.86 ± 3.16 | 0.201 |
| CSS Factor 4-Loneliness (0-14) | 4.47 ± 3.25 | 3.40 ± 2.97 | <0.001 |
| CSS Factor 5-Financial (0-8) | 3.74 ± 2.25 | 2.55 ± 2.12 | <0.001 |
| CSS Factor 6-Workload (0-6) | 3.95 ± 1.58 | 3.91 ± 1.61 | 0.773 |
| CSS Factor 7-Debt (0-4) | 0.80 ± 1.22 | 1.16 ± 1.38 | 0.002 |
| CSS Factor 8-Relationship Inoccupancy (0-4) | 1.75± 1.34 | 0.61 ± 1.09 | <0.0001 |
| CSS Factor 9-Family Health (0-6) | 0.78 ± 1.15 | 1.33 ± 1.67 | <0.001 |
| CSS Factor 10-Residence (0-4) | 1.51 ± 1.34 | 1.34 ± 1.29 | 0.179 |
| CSS Factor 11-Family (0-4) | 0.61 ± 0.92 | 0.54 ± 0.91 | 0.294 |
| CSS Factor 12-Ex-Partner (0-4) | 0.17 ± 0.56 | 0.29 ± 0.71 | 0.035 |
| CSS Factor 13 (0-4) | 1.10 ± 1.04 | 1.19 ± 1.15 | 0.566 |
| CSS Total Score (0-102) | 27.66 ± 14.14 | 28.49 ± 14.72 | 0.424 |
| BDI (0-63) | 17.71 ± 12.10 | 13.77 ± 10.75 | <0.001 |
| BAI (0-63) | 18.67 ± 13.93 | 15.09 ± 13.29 | 0.001 |
| PSS-14 (0-56) | 32.16 ± 7.38 | 29.89 ± 7.37 | <0.001 |
| Note: CSS: Chronic Stress Scale, BDI: Beck Depression Inventory, BAI: Beck Anxiety Inventory, PSS-14: Perceived Stress Scale-14, Mann-Whitney U test | | | |

Supplementary Table 6. Comparison of the sex groups for the CSS factors, CSS total score, BDI, BAI and PSS-14

| **Scale (Min-Max scores)** | **Female (Mean ± S.D.)** | **Male (Mean ± S.D.)** | **p** |
| --- | --- | --- | --- |
| CSS Factor 1- Partner (0-18) | 4.32 ± 4.84 | 3.67 ± 4.36 | 0.257 |
| CSS Factor 2 - Children (0-12) | 1.48 ± 2.67 | 0.90 ± 1.99 | 0.040 |
| CSS Factor 3-Work (0-12) | 4.13 ± 3.30 | 4.16 ± 3.25 | 0.885 |
| CSS Factor 4-Loneliness (0-14) | 4.17 ± 3.26 | 4.02 ± 3.07 | 0.826 |
| CSS Factor 5-Financial (0-8) | 3.40 ± 2.35 | 3.24 ± 2.13 | 0.430 |
| CSS Factor 6-Workload (0-6) | 4.02 ± 1.59 | 3.77 ± 1.58 | 0.096 |
| CSS Factor 7-Debt (0-4) | 0.87 ± 1.24 | 1.01 ± 1.38 | 0.387 |
| CSS Factor 8-Relationship Inoccupancy (0-4) | 1.40± 1.40 | 1.32 ± 1.32 | 0.600 |
| CSS Factor 9-Family Health (0-6) | 0.98 ± 1.40 | 0.92 ± 1.30 | 0.730 |
| CSS Factor 10-Residence (0-4) | 1.53 ± 1.35 | 1.27 ± 1.25 | 0.043 |
| CSS Factor 11-Family (0-4) | 0.67 ± 0.98 | 0.43 ± 0.75 | 0.018 |
| CSS Factor 12-Ex-Partner (0-4) | 0.21 ± 0.61 | 0.22 ± 0.63 | 0.826 |
| CSS Factor 13 (0-4) | 1.26 ± 1.13 | 0.87 ± 0.91 | <0.001 |
| CSS Total Score (0-102) | 28.74 ± 14.59 | 26.22 ± 13.63 | 0.059 |
| BDI (0-63) | 17.42 ± 11.96 | 14.31 ± 11.24 | 0.004 |
| BAI (0-63) | 19.38 ± 14.54 | 13.50 ± 11.15 | <0.001 |
| PSS-14 (0-56) | 32.17 ± 7.41 | 29.82 ± 7.28 | <0.001 |
| Note: CSS: Chronic Stress Scale, BDI: Beck Depression Inventory, BAI: Beck Anxiety Inventory, PSS-14: Perceived Stress Scale-14, Mann-Whitney U test | | | |

a.


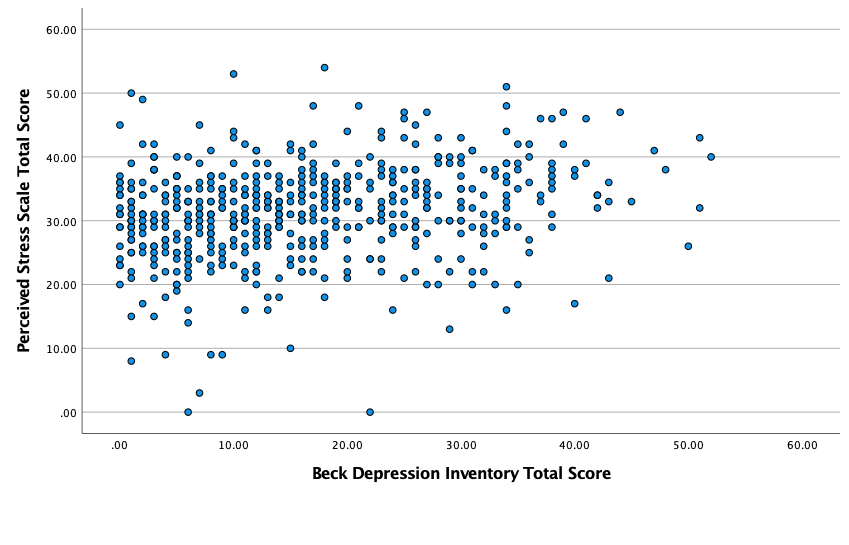


b.


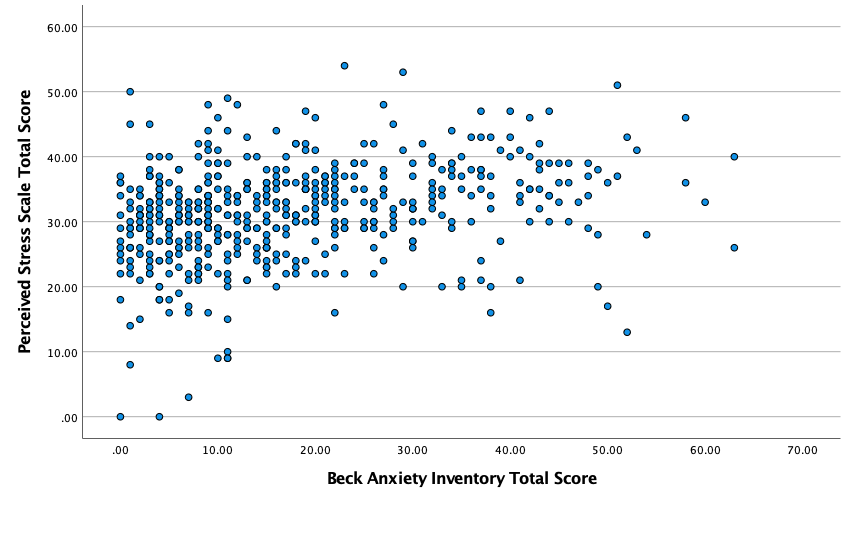


c.


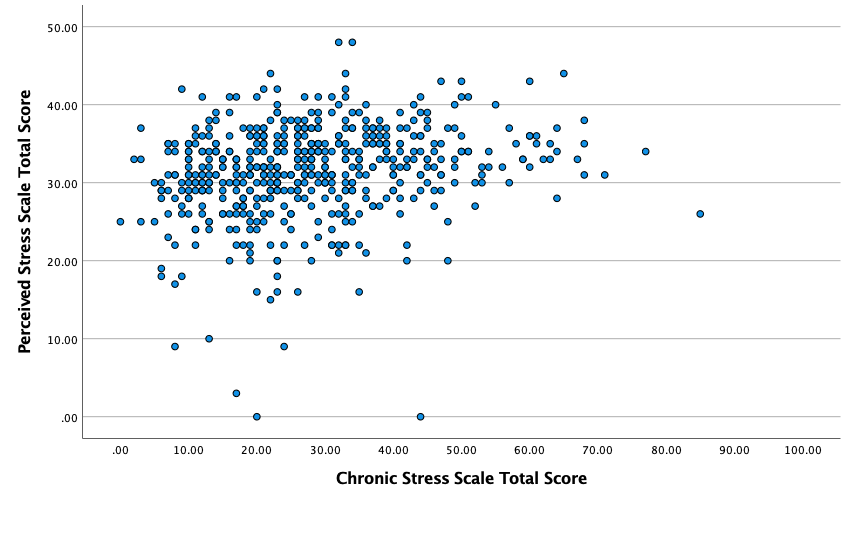


Supplementary Figure 1.Correlation of Perceived Stress Scale (PSS) with (a) Beck Depression Inventory (BDI), (b) Beck Anxiety Inventory (BAI) and (c) Chronic Stress Scale (CSS). PSS showed a weak correlation with BDI (r=0.236, p<0.001), BAI (r=0.28, p<0.001) and CSS (r=0.22, p<0.001)

**Appendix 1. Turkish Version of the CSS**

**Kronik Stres Ölçeği**

Şimdi size insanların yaşamında karşılaşabilecekleri bazı durumlar vereceğiz. Eğer bunlar doğru değil, bir miktar doğru veya sizin için tamamen doğru ise işaretleyiniz.

1. Aynı anda çok fazla şeyi yüklenmeye çalışıyorsunuz.

- Doğru değil
- Biraz doğru
- Çok doğru

1. Diğer insanlar gibi olmak için üzerinizde çok baskı var.

- Doğru değil
- Biraz doğru
- Çok doğru

1. Diğerlerinin sizden çok fazla beklentisi var.

- Doğru değil
- Biraz doğru
- Çok doğru

1. Kendinizin ya da çocuklarınızın ihtiyaçlarını almak için yeterli paranız yok.

- Doğru değil
- Biraz doğru
- Çok doğru

1. Uzun süreli borcunuz ya da krediniz var.

- Doğru değil
- Biraz doğru
- Çok doğru

1. Kiranız ya da kredi ödemeniz çok fazla.

- Doğru değil
- Biraz doğru
- Çok doğru

1. Seyahatlere çıkmak için yeterli paranız yok.

- Doğru değil
- Biraz doğru
- Çok doğru

1. Bir ev için peşin ödeme yapacak yeterli paranız yok.

- Doğru değil
- Biraz doğru
- Çok doğru

1. Birçok insana göre yapmanız gereken çok iş var.

- Doğru değil
- Biraz doğru
- Çok doğru

1. Danışmanınız sürekli işte ne yaptığınızı izliyor.

- Doğru değil
- Biraz doğru
- Çok doğru

1. İşinizi ya da kariyerinizi değiştirmek istiyorsunuz fakat yapabileceğinizi hissetmiyorsunuz.

- Doğru değil
- Biraz doğru
- Çok doğru

1. İşiniz çoğunlukla sizi ruhsal ve fiziksel olarak yorgun hisseder bir hale getiriyor.

- Doğru değil
- Biraz doğru
- Çok doğru

1. İşte daha başarılı olmak istiyorsunuz ancak işler yürümüyor.

- Doğru değil
- Biraz doğru
- Çok doğru

1. Yaptıklarınız için yeterli ödemeyi alamıyorsunuz.

- Doğru değil
- Biraz doğru
- Çok doğru

1. İşiniz sıkıcı ve tekrarlayıcı.

- Doğru değil
- Biraz doğru
- Çok doğru

1. İş arıyorsunuz ve aradığınızı bulamıyorsunuz.

- Doğru değil
- Biraz doğru
- Çok doğru

1. Partnerinizle birçok çelişen yönünüz var.

- Doğru değil
- Biraz doğru
- Çok doğru

1. İlişkiniz özgürlüğünüzü kısıtlıyor.

- Doğru değil
- Biraz doğru
- Çok doğru

1. Partneriniz sizi anlamıyor.

- Doğru değil
- Biraz doğru
- Çok doğru

1. Partnerinizin sizden çok fazla beklentisi var.

- Doğru değil
- Biraz doğru
- Çok doğru

1. İlişkinizde hak ettiğiniz değeri alamıyorsunuz.

- Doğru değil
- Biraz doğru
- Çok doğru

1. Partneriniz size yeterli ilgiyi göstermiyor.

- Doğru değil
- Biraz doğru
- Çok doğru

1. Partneriniz ilişkinize yeterli bağlılık göstermiyor.

- Doğru değil
- Biraz doğru
- Çok doğru

1. Bu ilişkiniz cinsel ihtiyaçlarınızı karşılayamıyor.

- Doğru değil
- Biraz doğru
- Çok doğru

1. Partneriniz her zaman ilişkiyi bitirmekle ya da ayrılmakla sizi tehdit ediyor.

- Doğru değil
- Biraz doğru
- Çok doğru

1. Herhangi bir zaman evlenip evlenmeyeceğinizi merak ediyorsunuz.

- Doğru değil
- Biraz doğru
- Çok doğru

1. Sizinle uyumlu birini bulmakta çok zorluk çekiyorsunuz.

- Doğru değil
- Biraz doğru
- Çok doğru

1. Eski eşinizle çok fazla çatışmanız var.

- Doğru değil
- Biraz doğru
- Çok doğru

1. Önceki evliliğinizden olan çocuklarınızı istediğiniz kadar göremiyorsunuz.

- Doğru değil
- Biraz doğru
- Çok doğru

1. Çok fazla yalnızsınız.

- Doğru değil
- Biraz doğru
- Çok doğru

1. Çocuklarınız olsun isterdiniz ama çocuklarınız yok.

- Doğru değil
- Biraz doğru
- Çok doğru

1. Çocuklarınızdan bir tanesi çok mutsuz görünüyor.

- Doğru değil
- Biraz doğru
- Çok doğru

1. Çocuklarınızın sizi dinlemediğini hissediyorsunuz.

- Doğru değil
- Biraz doğru
- Çok doğru

1. Çocuklarınızdan birinin davranışları sizin için ciddi bir endişe kaynağı.

- Doğru değil
- Biraz doğru
- Çok doğru

1. Bir ya da birden fazla çocuğunuz işte ya da okulda yeterince iyi yapamıyor.

- Doğru değil
- Biraz doğru
- Çok doğru

1. Çocuklarınız ev işlerine yardım etmez.

- Doğru değil
- Biraz doğru
- Çok doğru

1. Çocuklarınızdan bir tanesi ev dışında çok fazla zaman geçiriyor.

- Doğru değil
- Biraz doğru
- Çok doğru

1. Ev hanımı olmanın takdir edilmediğini hissediyorsunuz.

- Doğru değil
- Biraz doğru
- Çok doğru

1. Sosyal etkinliklere yalnız katılmak zorundasınız ancak istemiyorsunuz.

- Doğru değil
- Biraz doğru
- Çok doğru

1. Arkadaşlarınız kötü etkileyebilen kişilerdir.

- Doğru değil
- Biraz doğru
- Çok doğru

1. Yeterli arkadaşınız yok.

- Doğru değil
- Biraz doğru
- Çok doğru

1. En sevdiğiniz boş zaman aktivitelerine ayıracak zamanınız yok.

- Doğru değil
- Biraz doğru
- Çok doğru

1. Ailenizden çok uzakta yaşamak istiyorsunuz.

- Doğru değil
- Biraz doğru
- Çok doğru

1. Taşınmak istiyorsunuz ancak taşınamıyorsunuz.

- Doğru değil
- Biraz doğru
- Çok doğru

1. Yaşadığınız yer çok gürültülü ya da çok kirli.

- Doğru değil
- Biraz doğru
- Çok doğru

1. Aileniz çok uzakta yaşıyor.

- Doğru değil
- Biraz doğru
- Çok doğru

1. Ailenizden birisi ya da yakın bir arkadaşınızın uzun süredir devam eden bir hastalığı ya da bir engelliliği var.

- Doğru değil
- Biraz doğru
- Çok doğru

1. Sağlık durumu kötü ya da ölmek üzere olan bir ebeveyniniz, çocuğunuz ya da eş veya partneriniz var.

- Doğru değil
- Biraz doğru
- Çok doğru

1. Ailenizden birisinin alkol veya madde sorunu var.

- Doğru değil
- Biraz doğru
- Çok doğru

1. Uzun süredir devam eden bir sağlık problemi, yapmak istediğiniz şeyleri yapmanıza engel oluyor.

- Doğru değil
- Biraz doğru
- Çok doğru

1. Hemen hemen her gün yaşlanan bir ebeveyninize bakım veriyorsunuz.

- Doğru değil
- Biraz doğru
- Çok doğru
